# Replicating the real-world evidence methods available in human health to assess burden and outcomes for dogs with chronic kidney disease, their owners, and the veterinary healthcare system in the United States of America

**DOI:** 10.3389/fvets.2025.1502933

**Published:** 2025-02-21

**Authors:** Andrea Wright, Dean Taylor, Mollie Lowe, Sophie Barlow, James Jackson

**Affiliations:** ^1^Outcomes Research, Zoetis, Parsippany, NJ, United States; ^2^Adelphi Real World, Bollington, United Kingdom

**Keywords:** real-world, canine, chronic kidney disease, owner burden, disease management, cross-sectional study, animal health, veterinarian-owner communication

## Abstract

**Introduction:**

Chronic kidney disease (CKD) in canines is a progressive condition characterized by a gradual decline in kidney function. There are significant gaps in understanding how CKD is managed in canines and the full extent of its impact. This study aimed to characterize disease management of CKD and its impact on dogs, their owners and the veterinary healthcare system in the United States of America (United States).

**Methods:**

Data were drawn from the Adelphi Real World Canine CKD Disease Specific Programme™, a cross-sectional survey of veterinarians, pet owners and their dogs with CKD in the United States from December 2022 to January 2024. Veterinarians reported demographic, diagnostic, treatment, and healthcare utilization data, for dogs with CKD. Owners voluntarily completed questionnaires, providing data about their dog, as well as quality of life and work-related burden using the Dog Owners Quality of Life, and the Work Productivity and Activity Impairment questionnaires. Analyses were descriptive and Cohen’s Kappa was used to measure agreement between owners and veterinarians.

**Results:**

A total of 117 veterinarians provided data for 308 dogs, of which 68 owners also reported information. Discrepancies in recognizing symptoms of CKD in dogs, particularly excessive water consumption and urination, were identified between veterinary professionals and owners. Interventions for managing CKD in dogs focused on controlling symptoms and supporting kidney function through dietary modifications and medication. Owners of dogs with CKD reported minimal impact to overall work and activity impairment (10 and 14%, respectively). At diagnosis, 78.6% of dogs were International Renal Interest Society Stage I-II, and 21.5% were Stage III-IV. Regardless of CKD stage, owners strongly agreed that ownership provided them with emotional support and companionship. Regarding veterinary healthcare utilization, 95% of dogs were seen in general veterinary practices.

**Discussion:**

These findings emphasize the value of real-world evidence in enhancing our understanding of CKD in companion animals and informs future strategy for the real-world diagnosis and treatment of CKD. The results also provide insights to the potential burden experienced by owners of dogs with CKD.

## 1 Introduction

Chronic kidney disease (CKD) is a common renal disease in older dogs (1, 2), with an estimated prevalence of up to 3% in the general population (1–3) and up to 10% in hospitalized dogs (4). CKD is characterized by structural and/or functional impairment of one or both kidneys for at least 3 months that is irreversible and progressive (1, 4, 5). The International Renal Interest Society (IRIS) staging system defines 4-stages of CKD, ranging from stage I (mild loss of kidney function) to stage IV (end stage renal failure), which aids in diagnosis, management, and assessment of response to treatment (6).

In the early stages of the disease, increased urination and increased water consumption (polyuria and polydipsia, respectively) can occur due to the inability of the kidneys to regulate water homeostasis (2, 6). As the disease progresses, further symptoms develop such as loss of body condition, weight, and muscle mass, an unkempt appearance, hyporexia, anorexia, vomiting, lethargy, diarrhea, halitosis, azotemia, and ulcerative stomatitis and gastroenteritis, as well as electrolyte and acid–base imbalances (2–4, 7, 8). Disease progression varies, with factors such as anemia, low body condition score, proteinuria, hypertension, hypoalbuminemia, hyperphosphatemia, and IRIS stage playing key roles in prognosis (1, 2, 9–11). CKD may be diagnosed at a later stage, when renal damage is already severe and the prognosis is more guarded, due to limitations of renal function biomarkers, non-compliance with the recommended frequency of check-ups, the difficulty owners have in identifying early clinical signs, and their respective delay in seeking veterinary assistance (4, 6, 12, 13). It is thought that earlier diagnosis and treatment of clinical signs may slow progression and improve quality of life for canine patients (2).

Interventions for managing CKD in dogs focus on controlling symptoms and supporting kidney function. For example, the primary recommendation for management of CKD in dogs is dietary modification, with low-protein and phosphate-restricted diets being commonly recommended to reduce workload on the kidneys (14–16). In addition to this, fluid therapy and antiemetic use are recommended to maintain hydration and to treat vomiting and nausea, respectively (16). Similarly, other medications such as antihypertensive and antiproteinuric drugs can be prescribed to address potential CKD complications, such as systemic hypertension and renal proteinuria, respectively (17–20).

Currently, little is known about the burden experienced by owners of dogs with CKD, despite caregiver burden being a well-researched concept in human medicine. To date, studies have focused on the comparison between the burden carried by owners of ill pets and those with healthy pets, suggesting that increased stress, symptoms of anxiety and depression, and reduced quality of life were more prevalent in the former (21–23). These findings were, however, limited due to the lack of disease-specific data included in each study. It is reasonable to assume that the need for frequent veterinarian visits, testing and diagnostic exams, dietary modifications, lifestyle changes and medication administration could impact an owner’s time, mental load, and finances. However, data on this issue is still scarce in the literature, and our understanding of the owners’ burden related to specific canine diseases, such as CKD, remains limited.

Although randomized clinical trials (RCTs) are crucial to determine the effects of treatment in controlled settings, they often lack external validity (24, 25). Real-world evidence (RWE) can externally validate beyond RCT populations, increasing understanding of the clinical management of diseases compared to guideline recommendations (24–26), as well as the benefits and risks of treatments within a real-world patient population (27).

The Adelphi Real World Disease Specific Program™ (DSP) is a validated source of real-world data (28–31) and has been used to generate RWE in over 100 human disease areas. Findings from the DSP have furthered our understanding of disease management and treatment patterns (32, 33), patient quality of life (34), physician-patient disconnect (35), caregiver burden (36), and health-care resource utilization (37).

Although RWE generation is routinely used in human health, there is a lack of similar research in animal health, based on information provided by veterinarians and pet owners. To the authors’ knowledge, this study is the first of its kind in the animal health space, aiming to apply the DSP methodology to demonstrate the relevance of RWE in assessing the impact of canine CKD on affected dogs, their owners and the veterinary healthcare system.

## 2 Methods

### 2.1 Study design

Data were extracted from the Adelphi Real World Canine CKD DSP, a large cross-sectional survey, with retrospective data collection, of veterinarians, owners and their dogs with CKD and conducted in the United States of America (United States) between December 2022 and January 2024. The DSP methodology has been previously described (28, 29) and validated (30) and has shown to be representative and consistent over time (31).

Veterinarians were recruited to participate in the DSP following completion of a short screening questionnaire. Veterinarians were eligible if they were personally responsible for treatment decisions and management of a minimum of 1–4 dogs with CKD per month. Veterinarian participation was financially incentivized, with reimbursement upon survey completion according to fair market research rates.

Dogs were eligible for inclusion if they had been examined by a veterinarian, were at least 3 years old, had a confirmed diagnosis of CKD, and their primary caregiver was at least 18 years of age.

### 2.2 Veterinarian-reported data

Upon recruitment, veterinarians completed a survey which included demographic data. Following this, veterinarians were invited to complete a questionnaire for their next five consecutively consulting canine patients, who visited them for routine care and met the eligibility criteria. The veterinarian-reported questionnaire contained questions on canine patient demographics, consultation history, diagnostic tests and monitoring of CKD, clinical signs, IRIS stage, prescribed CKD treatment, satisfaction and adherence to prescribed treatment, concomitant conditions, emergency visits, hospitalizations and other recommendations given.

Completion of the veterinarian-reported questionnaires was undertaken through consultation of existing canine patient clinical records, as well as the judgment and diagnostic skills of the respondent veterinarian, consistent with decisions made in routine real-world clinical practice.

### 2.3 Owner-reported data

The owner of each canine patient for whom the veterinarian completed a veterinarian-reported questionnaire was invited to voluntarily complete an owner-reported questionnaire. Owner-reported questionnaires were completed independently of the veterinarian and returned in a sealed envelope, ensuring the owners responses were kept confidential.

Owner-reported questionnaires contained questions on owner demographics, insurance details, positive and negative aspects of owning a dog with CKD using the Dog Owner Quality of Life (DOQOL) questionnaire (38), work-related burden using the Work Productivity and Activity Impairment (WPAI) questionnaire (39) and information about their dog’s condition, including clinical signs and their severity, level of satisfaction with their dog’s treatment, and other recommendations.

The DOQOL is a 10-item questionnaire that includes statements relating to three different factors: emotional wellbeing, social and physical activities, and stress and responsibility of dog ownership (38). Responses to each item are measured using a 7-point Likert scale which includes the following choices: strongly disagree, mostly disagree, somewhat disagree, neither agree or disagree, somewhat agree, mostly agree, and strongly agree (38). Results are reported on an item-by-item basis.

The WPAI measured impairment using four domains: absenteeism (work time missed), presenteeism (impairment at work), overall work impairment, and overall activity impairment (activities that do not include work) over the 7 days prior to survey (39). Scores are expressed as impairment percentages and range from 0 to 100, with higher scores indicating greater impairment (39).

### 2.4 Ethical considerations

Using a checkbox, owners provided consent to take part in the survey. No potential identifiable information was extracted during the study, so neither the veterinarians nor the pet owners could be directly identified. In addition, the generated data were aggregated prior to analysis and publication.

Data collection was undertaken in line with European Pharmaceutical Marketing Research Association guidelines and, therefore, did not require ethics committee approval (40). In addition, each survey was performed in full accordance with relevant legislation at the time of data collection, including the US Health Insurance Portability and Accountability Act 1996 (41), and Health Information Technology for Economic and Clinical Health Act legislation (Health Information Technology (42).

### 2.5 Data analysis

Mean and standard deviation (SD) were calculated for continuous variables, and frequency and percentages for categorical variables.

Cohen’s Kappa was used to measure agreement of owner-reported and veterinarian-reported clinical signs. The Kappa statistic (*κ*) was determined between −1 and 1, depending on whether the level of agreement was interpreted as poor (<0.00), slight (0.00–0.20), fair (0.21–0.40), moderate (0.41–0.60), substantial (0.61–0.80), or almost perfect (0.81–1.00) (43).

Descriptive analyses were performed on the positive and negative aspects of owning a dog with CKD. Dogs were grouped into two groups based on their current IRIS stage: early (I and II) and late (III and IV) stages.

All descriptive analyses were conducted in UNICOM Intelligence Survey Reporter version 7.5.1 (44) and all statistical analyses were conducted in Stata v18 (45). Missing data were not considered and, therefore, not all patients had information regarding all variables, leading to each one being described and analyzed separately. A significance level of *p* < 0.05 was used for all statistical analyses.

## 3 Results

### 3.1 Sample

A total of 117 veterinarians from the United States participated in this study: 13 from the Northwest, 23 from the Midwest, 39 from the South, and 22 from the West. For the remaining 20 veterinarians, region was not stated. Most veterinarians had qualified between 1986 and 2019 and provided data on a total of 308 dogs with CKD. All owners were offered the chance to complete an owner-reported questionnaire, of which 68 agreed. Whenever relevant, the results are presented on a matched basis, associating data from veterinarian questionnaires and owner questionnaires for the same dogs. All other data is presented for the full dataset, unless stated otherwise (Table 1).

**Table 1:** Sample.

Sample	
Total number of veterinarians and owners, *n*	
Veterinarians	117
Owners	68
Total number of completed forms, *n*	
Veterinarian surveys	100
Veterinarian-reported questionnaires	308
Owner-reported questionnaires	68
Veterinary speciality, *n* (%):	(*n* = 117)
Veterinarian	111 (94.9)
Veterinary licensed technician	3 (2.6)
Emergency veterinarian	1 (<1.0)
Veterinary assistant	1 (<1.0)
Other veterinary professional	1 (<1.0)
Veterinarian year of qualification, *n*, (%):	(*n* = 100)
Before 1986	6 (6.0)
1986–1998	26 (26.0)
1999–2008	41 (41.0)
2009–2019	26 (26.0)
After 2019	1 (1.0)
Location of veterinary practice, *n*, (%):	(*n* = 117)
Northeast	13 (11.1)
Midwest	23 (19.7)
South	39 (33.3)
West	22 (18.8)
Not stated	20 (17.1)

### 3.2 Canine CKD clinical characteristics and disease management

Of the 308 dogs included in this analysis, 173 were female, and mean (SD) age was 11.4 (3.4) years. Weight groupings were defined via the American Kennel Club guidelines and body condition groupings were taken from the World Small Animal Veterinary Association Body Condition Score Chart. Guidelines for weight groupings of mixed breed dogs were unavailable, which prevented 53 dogs being included in the weight analysis. Weight groupings for 255 dogs showed that 18% (*n* = 46) were underweight, 43% (*n* = 109) had ideal weight, and 39% (*n* = 100) were overweight. Body condition scores for 253 dogs showed 21% (*n* = 53) were below the ideal body condition score, 53% (*n* = 133) were in ideal body condition score and 27% (*n* = 67) were above the ideal body condition score (Table 2).

**Table 2:** Dog demographics.

Dog demographics	
Sex, *n* (%)	(*n* = 308)
Male	135 (43.8)
Female	173 (56.2)
Age, years	(*n* = 308)
Mean (SD)	11.4 (3.4)
Min, Max	3.0, 19.0
Weight^a^, *n* (%)	(*n* = 255)
Underweight	46 (18.0)
Ideal weight	109 (42.7)
Overweight	100 (39.2)
Body condition score^b^, *n* (%)	(*n* = 253)
Below ideal	53 (20.9)
Ideal	133 (52.6)
Above ideal	67 (26.5)

SD, standard deviation. ^a^Weight groupings were taken from the American Kennel Club, mixed breeds were removed; ^b^Groupings taken from the World Small Animal Veterinary Association Body Condition Score Chart.

### 3.3 IRIS stage

IRIS stage data were available for the full dataset (*n* = 308). At CKD diagnosis, 50% (*n* = 154) of all dogs were at IRIS stage II, with 29% (*n* = 88) at IRIS Stage I, 18% (*n* = 55) at IRIS Stage III and 4% (*n* = 11) at IRIS Stage IV. At the time of survey completion, the mean (SD) time since diagnosis (*n* = 295) was 11.7 (15.6) months and the proportion of dogs at IRIS stages I, II, III and IV was 21% (*n* = 66), 49% (*n* = 150), 21% (*n* = 66) and 8% (*n* = 26), respectively. Since first diagnosis, 79% (*n* = 244) of dogs had not progressed in IRIS stage (Table 3).

**Table 3:** International renal interest society (IRIS) staging.

IRIS staging	Total canine CKD patients
IRIS stage at diagnosis, *n* (%)	(*n* = 308)
Stage I	88 (28.6)
Stage II	154 (50.0)
Stage III	55 (17.9)
Stage IV	11 (3.6)
Months since diagnosis,	(*n* = 295)
Mean (SD)	11.7 (15.6)
Min, Max	0.0, 84.0
IRIS stage at the time of the survey, *n* (%)	(*n* = 308)
Stage I	66 (21.4)
Stage II	150 (48.7)
Stage III	66 (21.4)
Stage IV	26 (8.4)
Disease progression from diagnosis to time of survey?^a^, *n* (%)	(*n* = 308)
Yes (progressive)	64 (20.8)
No (non-progressive)	244 (79.2)

^aDefined as an increase in IRIS staging from the stage at initial diagnosis to the current stage. CKD, chronic kidney disease; IRIS, International Renal Interest Society; SD, standard deviation.^

### 3.4 Comorbidities

The mean (SD) number of comorbidities experienced per dog was 2.0 (1.6), with 18% (*n* = 56) of dogs having no comorbidities. The most reported comorbidities were periodontal disease (37%), degenerative joint disease (33%), and mitral valve disease (16%; Table 4).

**Table 4:** Comorbidities.

Comorbidities	Total canine CKD patients
Total number of comorbidities	(*n* = 308)
Mean (SD)	2.0 (1.6)
Min, Max	0.0, 8.0
Most frequent comorbidities, n (%)	(*n* = 308)
Periodontal disease	114 (37.0)
Degenerative joint disease	100 (32.5)
Mitral valve disease	48 (15.6)
No comorbidities	56 (18.2)

SD, standard deviation.

### 3.5 Clinical signs

Data on clinical signs were available for 253 dogs. Veterinarians reported that 42% of dogs experienced increased water consumption, 37% experienced increased or excessive urination, and 25% experienced unexplained weight loss (Table 5; Supplementary Table S1).

**Table 5:** Clinical signs.

Symptoms	Total canine CKD patients
Most frequent veterinarian-reported clinical signs, *n* (%)	(*n* = 253)
Increased/excessive water consumption	107 (42.3)
Increased/excessive urination	94 (37.2)
Unexplained weight loss	64 (25.3)
Veterinarian-reported total number of clinical signs per dog,	(*n* = 59)
Mean (SD)	3.0 (3.1)
Min, Max	0.0, 11.0
Most frequent veterinarian-reported clinical signs (matched base)^a^, *n* (%)	(*n* = 59)
Weakness/fatigue	21 (35.6)
Increased/excessive urination	19 (32.2)
Increased/excessive water consumption	17 (28.8)
Unexplained weight loss	17 (28.8)
No clinical signs	21 (35.6)
Veterinarian-reported most problematic clinical signs, *n* (%)	(*n* = 33)
Weakness/fatigue	10 (30.3)
Unexplained weight loss	10 (30.3)
Dehydration	9 (27.3)
Owner-reported total number of clinical signs per dog,	(*n* = 59)
Mean (SD)	2.8 (2.8)
Min, Max	0.0, 11.0
Most frequent owner-reported clinical signs, *n* (%)	(*n* = 59)
Increased/excessive water consumption	26 (44.1)
Loss of appetite	25 (42.4)
Increased/excessive urination	24 (40.7)
Weakness/fatigue	24 (40.7)
Lethargy	24 (40.7)
No clinical signs	8 (13.6)
Owner-reported most problematic clinical signs, *n* (%)	(*n* = 33)
Weakness/fatigue	14 (42.4)
Hyporexia/reduced appetite	14 (42.4)
Lethargy	13 (39.4)

Clinical signs disconnect^b^	Cohen’s Kappa analysis		
	κ	(SE)	*p*-value
Osteoporosis	−0.03	0.10	0.3928
Urinary tract infections	−0.03	0.10	0.3928
Halitosis	−0.02	0.13	0.4501
Blood in urine	−0.02	0.12	0.4251
Increased/excessive water consumption	0.04	0.12	0.3842
Increased/excessive urination	0.09	0.13	0.2355
Diarrhea	0.11	0.06	0.0289
Gastroenteritis	0.21	0.11	0.0290
Problems with vision	0.21	0.12	0.0406
Lethargy	0.22	0.12	0.0389
Hyporexia	0.26	0.12	0.0132
Vomiting	0.26	0.12	0.0201
No clinical signs	0.27	0.11	0.0061
Dehydration	0.30	0.11	0.0025
Weakness/fatigue	0.32	0.13	0.0068
Depression	0.37	0.13	0.0020
Unexplained weight loss	0.44	0.13	0.0003
Anorexia	0.44	0.13	0.0004
Unkempt appearance	0.45	0.13	0.0003
Pale gums	0.66	0.12	0.0001

CKD, chronic kidney disease; SD, standard deviation; SE, standard error. ^a^Data are presented on a matched base, meaning data from the veterinarian completed questionnaire and owner reported questionnaire are presented together, for the same dogs. ^b^Defined using Cohen’s Kappa analysis of agreement.

Owners of dogs with CKD also reported on clinical signs, resulting in 59 dogs having matched-base data available from veterinarians and owners (Table 5).

The mean (SD) number of clinical signs was 3.0 (3.1) and 2.8 (2.8) for veterinarians and owners, respectively. Both veterinary professionals and owners reported weakness/fatigue, increased urination, and increased water consumption as common clinical manifestations. Veterinarians also reported unexplained weight loss as a common finding, while owners listed loss of appetite and lethargy as the other most frequent abnormalities. Veterinary professionals and owners reported that 36% (*n* = 21) and 14% (*n* = 8) of dogs, respectively, showed no clinical signs suggestive of CKD (Table 5).

Cohen’s Kappa analysis identified a poor level of agreement between veterinarians and owners for osteoporosis and urinary tract infections, halitosis, and blood in urine. A slight level of agreement was identified between veterinarians and owners for increased/excessive water consumption (*κ* = 0.04), increased/excessive urination (*κ* = 0.09) and diarrhea (*κ* = 0.11). A fair level of agreement was identified for gastroenteritis (*κ* = 0.21), problems with vision (*κ* = 0.21), lethargy (*κ* = 0.22), hyporexia (*κ* = 0.26), vomiting (*κ* = 0.26), dehydration (*κ* = 0.30), weakness/fatigue (*κ* = 0.32), depression (*κ* = 0.37), and no clinical signs (*κ* = 0.27). Unexplained weight loss (*κ* = 0.44), anorexia (*κ* = 0.44) and unkempt appearance (*κ* = 0.45) had moderate levels of agreement between veterinarians and owners. There was a substantial level of agreement between veterinarians and owners for pale gums (*κ* = 0.66).

**Figure 1:**
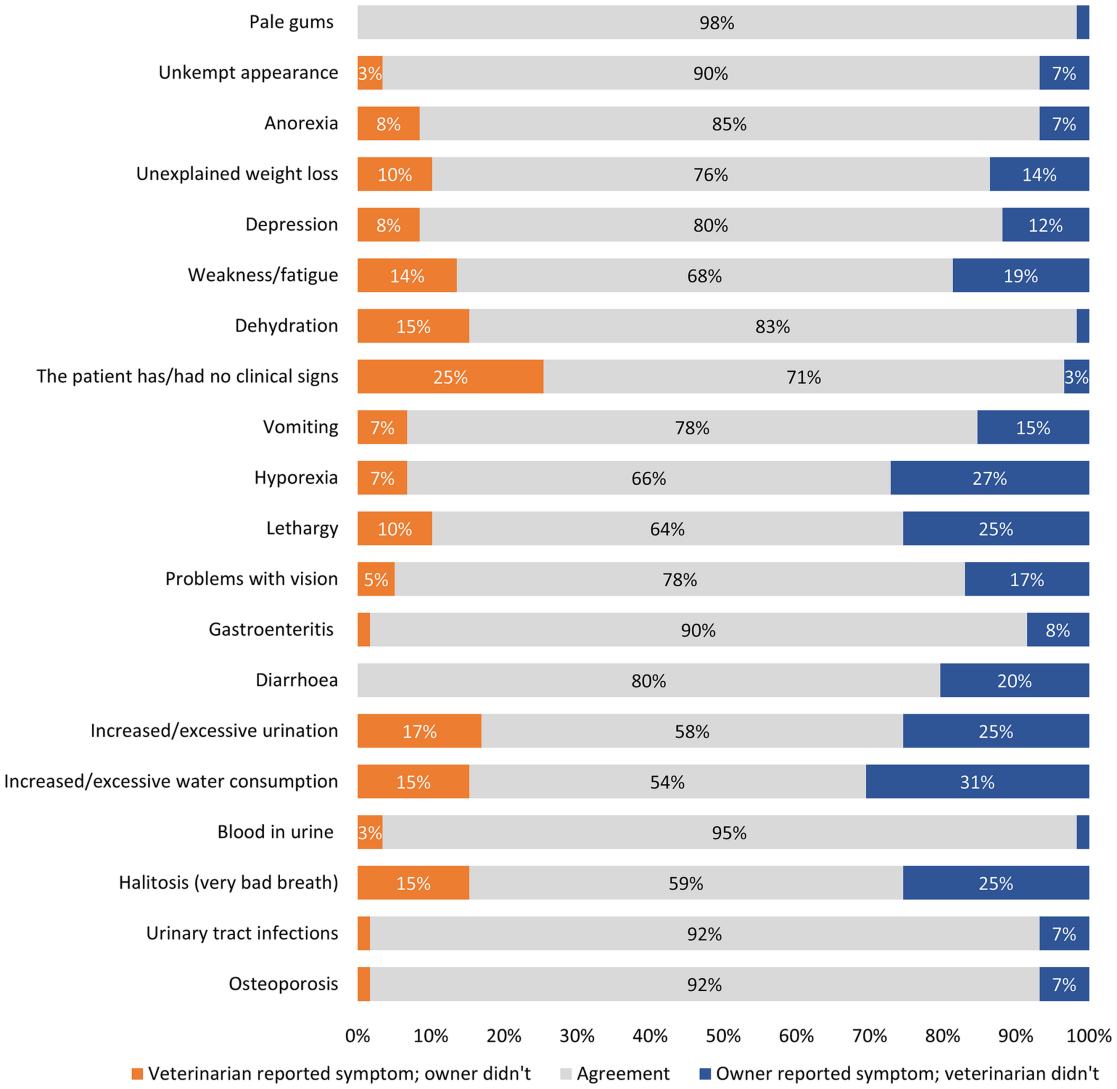
Level of agreement between veterinarian-reported and owner-reported clinical signs in dogs with CKD. *Please note that data points of less than 3% have been removed.

Owners reported clinical signs when veterinarians did not more often than the opposite. This was observed in the following clinical signs: osteoporosis, urinary tract infections, halitosis, increased water consumption, increased urination, diarrhea, gastroenteritis, problems with vision, lethargy, hyporexia, vomiting, weakness/fatigue, depressed mood, unexplained weight loss, unkempt appearance, and pale gums (Figure 1). In contrast, veterinarians reported more frequently dehydration, anorexia, blood in urine, and no clinical signs when owners did not, compared to the inverse situation (Figure 1).

Data on veterinary and owner perceptions of the most problematic clinical signs were available on a matched base (*n* = 33): weakness/fatigue was reported for 30% of dogs by veterinarian professionals and for 42% of dogs by owners. Veterinarian professionals also recorded unexplained weight loss (30%) and dehydration (27%) as the other most problematic clinical signs for dogs, whereas owners felt that hyporexia (42%) and lethargy (39%) were the most challenging.

### 3.6 CKD treatment

Data on treatment status is summarized in Table 6. Veterinarian-reported treatment status was available for 253 dogs and, among these, 36% (*n* = 90) were receiving treatment at the time of survey and 54% (*n* = 137) had never received treatment. Of the 90 dogs receiving treatment at the time of survey, the most prescribed therapies were maropitant (34%), subcutaneous fluid therapy (23%), aluminium hydroxide (18%), and capromorelin (18%). Other treatments received at the time of survey included benazepril (17%), enalapril (13%), omeprazole (12%), and telmisartan (11%). Treatment was initiated based on both the presence of clinical signs and test results in 71% (*n* = 64) of dogs, and the mean (SD) treatment duration was 7.3 (10.7) months (*n* = 80) at the time of survey. The most common issue with current treatment was progressive CKD (reported by 22% of veterinarians). Of 87 owners with veterinarian-perceived owner compliance, 28% were deemed to be completely adherent, 44% mostly adherent, 22% somewhat adherent, 5% little adherent, and 2% not adherent at all. A total of 30 owners reported information on adherence and medication administration, of which 80% stated they were always adherent, with 37 and 27% further reporting that administering the medication was easy or very easy, respectively. Of 6 owners who had difficulty administering medication to their dogs, 50% justified it by stating that their dog did not like the medication.

**Table 6:** Treatment.

Treatment	Total canine CKD patients
Treatment status, *n* (%)	(*n* = 253)
Never prescribed treatment	137 (54.2)
Previously prescribed treatment, not on treatment at survey	26 (10.3)
Prescribed treatment at survey	90 (35.6)
Top 10 treatments received at survey, *n* (%)	(*n* = 90)
Maropitant	31 (34.4)
Subcutaneous fluid therapy	21 (23.3)
Aluminum hydroxide	16 (17.8)
Capromorelin	16 (17.8)
Benazepril	15 (16.7)
Enalapril	12 (13.3)
Omeprazole	11 (12.2)
Telmisartan	10 (11.1)
Mirtazapine	9 (10.0)
Amlodipine	9 (10.0)
Factors influencing therapeutic decision at survey, *n* (%)	(*n* = 90)
Symptoms only	11 (12.2)
Test results only	15 (16.7)
Both symptoms and test results	64 (71.1)
Duration of treatment at survey, months	(*n* = 80)
Mean (SD)	7.4 (10.7)
Min, Max	0.0, 46.0
Issues with treatment at survey, *n* (%)	(*n* = 90)
CKD progression	20 (22.2)
Patient compliance issues	13 (14.4)
Owner compliance issues	12 (13.3)
No issues	24 (26.7)
Veterinarian-perceived compliance, *n* (%)	(*n* = 87)
Completely adherent	24 (27.6)
Mostly adherent	38 (43.7)
Somewhat adherent	19 (21.8)
A little adherent	4 (4.6)
Not at all adherent	2 (2.3)
Owner-perceived adherence, *n* (%)	(*n* = 30)
Always	24 (80.0)
Often	5 (16.7)
Sometimes	0 (0.0)
Rarely	0 (0.0)
Never	1 (3.3)
Reason for non-adherence, *n* (%)	(*n* = 6)
“My dog does not like to take medication”	3 (50.0)
“Medication does not have the desired effect”	1 (16.7)
“Frequency of administration is too high”	1 (16.7)
“I am worried that the dose is too high”	1 (16.7)
“Administration is difficult”	1 (16.7)
“I do not know enough about my dog’s CKD”	1 (16.7)
Medication Administration, *n* (%)	(*n* = 30)
Very difficult	0 (0.0)
Difficult	6 (20.0)
Neutral	5 (16.7)
Easy	11 (36.7)
Very easy	8 (26.7)

CKD, chronic kidney disease; SD, standard deviation.

### 3.7 Other interventions

Overall, data regarding veterinarian-recommended changes were available for 253 dogs. Among these, a renal diet was recommended to 74%, increased water intake to 48%, and adding water to kibble to 34% (Table 7; Supplementary Table S1).

**Table 7:** Other recommendations.

Other recommendations	Total canine CKD patients
Most frequent veterinarian-recommended changes, *n* (%)	(*n* = 253)
Veterinary prescribed renal diet	186 (73.5)
Increase water intake	121 (47.8)
Add water to kibble	85 (33.6)
Most frequent veterinarian-recommended changes (matched base)^a^, *n* (%)	(*n* = 60)
Veterinary prescribed renal diet	49 (81.7)
Increase water intake	32 (53.3)
Avoid foods high on sodium	25 (41.7)
No recommended changes	3 (5.0)
Veterinarian-reported implemented changes, *n* (%)	
Use veterinary prescribed renal diet, *n* (%)	39 (79.6) (*n* = 49)
Increase water intake, *n* (%)	29 (90.6) (*n* = 32)
Avoid food high in sodium, *n* (%)	24 (96.0) (*n* = 25)
Most frequent owner-reported recommended changes, *n* (%)	(*n* = 60)
Veterinary prescribed renal diet	53 (88.3)
Increase water intake	34 (56.7)
Avoid foods high on sodium	30 (50.0)
No recommended changes	0 (0.0)
Owners who have implemented changes, *n* (%)	
Use veterinary prescribed renal diet, *n* (%)	42 (79.2) (*n* = 53)
Increase water intake, *n* (%)	25 (73.5) (*n* = 34)
Avoid food high in sodium, *n* (%)	22 (73.3) (*n* = 30)

CKD, chronic kidney disease. ^a^Data are presented on a matched base, meaning data from the veterinarian completed questionnaire and owner reported questionnaire are presented together, for the same dogs.

Matched-base data on dietary changes were available for 60 dogs. Veterinarians and owners reported that the dogs were prescribed a renal diet (82 and 88%, respectively), recommended to increase water intake (53 and 57%, respectively), and advised to avoid foods high in sodium (42 and 50%, respectively). The implementation of these recommendations were also evaluated: veterinarians and owners reported implementing a renal diet in 80 and 79%, respectively, increasing water intake in 91 and 74%, respectively, and avoiding foods high in sodium in 96 and 73%, respectively (Table 7).

### 3.8 Experience of owning a dog with CKD

Of the 68 owners who completed the questionnaire, the majority were female (71%), with a mean (SD) age of 51.5 (16.1) years and among those for whom employment status (*n* = 67) was available, 52% were in full-time employment (Table 8). Of all owners who responded, 8% (*n* = 5) had pet insurance that provided coverage for their dog’s CKD.

**Table 8:** Owner demographics.

Owner demographics	Total canine CKD patients
Sex, *n* (%)	(*n* = 68)
Male	20 (29.4)
Female	48 (70.6)
Age, years	(*n* = 67)
Mean (SD)	51.5 (16.1)
Min, Max	22.0, 86.0
Owner age groups, *n* (%)	(*n* = 67)
<30, (%)	7 (10.4)
30–39, (%)	13 (19.4)
40–49, (%)	8 (11.9)
50–59, (%)	13 (19.4)
60–69, (%)	18 (26.9)
≥70, (%)	8 (11.9)
Highest level of education, *n* (%)	(*n* = 68)
Elementary school	0 (0.0)
Middle and high school	17 (25.0)
College or associate degree	29 (42.6)
Undergraduate degree	11 (16.2)
Graduate degree	11 (16.2)
Employment status, *n* (%)	(*n* = 67)
Full-time	35 (52.2)
Part-time	7 (10.4)
Unemployed	3 (4.5)
Retired	18 (26.9)
Student	1 (1.5)
Not working through choice	2 (3.0)
Long-term sick leave	1 (1.5)
Pet insurance, *n* (%)	(*n* = 64)
Yes	5 (7.8)
No	59 (92.2)

CKD, chronic kidney disease; SD, standard deviation.

### 3.9 Most problematic clinical signs for owners

Matched-base data regarding the most challenging clinical signs according to the owners’ perspective (*n* = 33) are reported in Table 9. Veterinarians reported weakness/fatigue as the most problematic in 46% (*n* = 15) of cases. Increased urination (39%), unexplained weight loss (30%), and lethargy (30%) were also reported by veterinarians as difficult clinical signs for the owners’ management. Owners reported hyporexia, increased urination and weakness/fatigue as the most challenging to care for in 64, 42 and 39% of cases, respectively.

**Table 9:** Most problematic clinical signs for the owner.

Most troublesome clinical signs for the owner	Total canine CKD patients
Veterinarian-reported most problematic clinical signs for owner, *n* (%)	(*n* = 33)
Weakness/fatigue	15 (45.5)
Increased/excessive urination	13 (39.4)
Unexplained weight loss	10 (30.3)
Lethargy	10 (30.3)
Owner-reported most problematic clinical signs for owner, *n* (%)	(*n* = 33)
Hyporexia/reduced appetite	21 (63.6)
Increased urination	14 (42.4)
Weakness/fatigue	13 (39.4)

CKD, chronic kidney disease.

### 3.10 Owner work productivity and activity impairment

Work Productivity and Activity Impairment (WPAI) scores are reported in Table 10. Mean (SD) presenteeism (impairment at work) was 9% (13.8%), absenteeism (work time missed) was 2% (9.9%), and overall work impairment was 10% (17.4%). Mean (SD) overall activity impairment (activities that do not include work) was 14% (17.2%).

**Table 10:** Work productivity and activity impairment (WPAI).

	Total canine CKD patients
WPAI: Overall work impairment, %	(*n* = 25)
Mean (SD)	10.3 (17.4)
Min, Max	0.0, 65.0
WPAI: Presenteeism, %	(*n* = 27)
Mean (SD)	8.5 (13.8)
Min, Max	0.0, 60.0
WPAI: Absenteeism, %	(*n* = 26)
Mean (SD)	2.4 (9.9)
Min, Max	0.0, 50.0
WPAI: Activity impairment, %	(*n* = 47)
Mean (SD)	14.0 (17.2)
Min, Max	0.0, 70.0

WPAI, work productivity and activity impairment; CKD, chronic kidney disease; SD, standard deviation.

### 3.11 Positive and negative aspects of owning a dog with CKD

**Figure 2:**
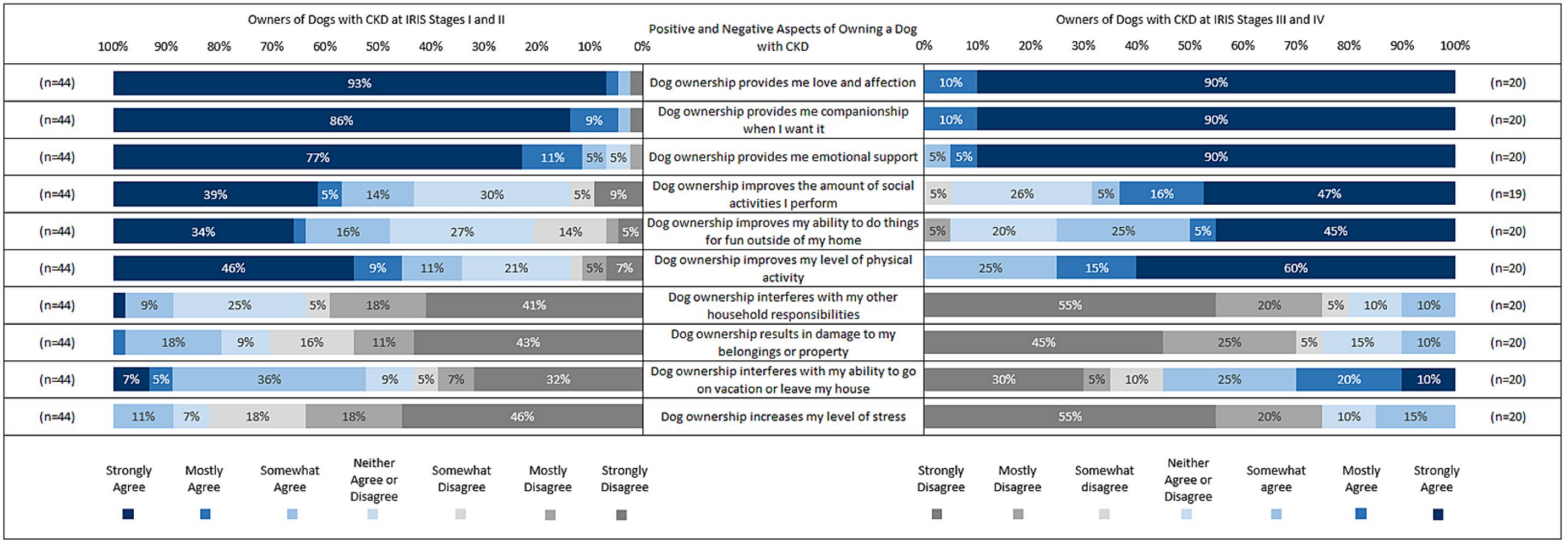
Positive and negative aspects of owning a dog with CKD by IRIS stage. *Please note that data points of less than 3% have been removed.

The positive and negative aspects of owning a dog with CKD were also explored based on IRIS Stage, so the sample was divided into IRIS Stage I-II (*n* = 44) and IRIS Stage III-IV (*n* = 20; Figure 2). Owners reported their level of agreement with each statement, ranging from strongly agree to strongly disagree.

In relation to the emotional wellbeing aspects of dog ownership, owners of dogs at IRIS I-II reported agreement that dog ownership provided them with love and affection (98%), companionship when wanted (98%), and emotional support (93%), with 93, 86 and 77% reporting strong agreement, respectively. All owners of dogs at IRIS III-IV agreed that dog ownership provides them with love and affection, companionship when wanted, and emotional support, with 90% reporting strong agreement with each item, respectively.

In relation to the social and physical aspects of dog ownership, owners of dogs at IRIS I-II reported agreement that dog ownership improves the amount of social activities they do (57%), improves their ability to do things for fun outside the house (52%), and improves their level of physical activity (66%), with 39, 34, and 46% reporting strong agreement, respectively. Owners of dogs at IRIS III-IV reported agreement that dog ownership improves the amount of social activities they perform (69%), improves their ability to do things for fun outside the house (75%), and improves their level of physical activity (100%), with 47, 45, and 60% reporting strong agreement, respectively.

In relation to stress and responsibilities of dog ownership, owners of dogs at IRIS I-II reported disagreement that dog ownership interferes with their household responsibilities (64%), results in damage to their belongings/property (71%), interferes with their ability to go on vacation or leave their home (43%), and increases their level of stress (82%), with 41, 43, 32, and 46% reporting strong disagreement, respectively. Owners of dogs at IRIS III-IV reported disagreement that dog ownership interferes with their household responsibilities (80%), results in damage to their belongings/property (75%), interferes with their ability to go on vacation or leave their home (45%), and increases their level of stress (75%), with 55, 45, 30, and 55% reporting strong disagreement, respectively.

### 3.12 Veterinary healthcare utilization

Data on veterinary healthcare resource utilization is summarized in Table 11. Most dogs were enrolled at general practice veterinary clinics and hospitals (95%) and the remainder were enrolled at private specialty referral veterinary clinics/hospitals, academic specialty referral veterinary clinics/hospitals, veterinary emergency clinics/hospitals and telemedicine.

**Table 11:** Veterinary healthcare resource utilization.

Number of appointments relating to CKD in the last 12 month	Total canine CKD patients
Number of appointments with all professional veterinarians	(*n* = 253)
Mean (SD)	3.9 (6.5)
Min, Max	1.0, 63.0
Veterinarians	(*n* = 248)
Mean (SD)	3.0 (2.5)
Min, Max	1.0, 16.0
Other veterinary professionals	(*n* = 41)
Mean (SD)	5.2 (10.1)
Min, Max	1.0, 48.0
Veterinary practice type for the appointment, *n* (%)	(*n* = 253)
General practice veterinary clinic/hospital	241 (95.3)
Private speciality referral veterinary clinic/hospital	5 (2.0)
Academic speciality referral veterinary clinic/hospital	2 (0.8)
Veterinary emergency clinic/hospital	2 (0.8)
Telemedicine	3 (1.2)
Number of tests used to aid diagnosis of CKD	(*n* = 253)
Mean (SD)	8.3 (4.0)
Min, Max	0.0, 17.0
Most frequent tests to aid diagnosis of CKD, *n* (%)	(*n* = 253)
Serum creatinine	234 (92.5)
Blood urea nitrogen	218 (86.2)
Complete blood count	190 (75.1)
Number of tests to monitor CKD, *n* (%)	(n = 240)
Mean (SD)	7.3 (4.8)
Min, Max	0.0, 20.0
Most frequent tests to monitor CKD, *n* (%)	(*n* = 240)
Serum creatinine	194 (80.8)
Blood urea nitrogen	176 (73.3)
Clinical signs and physical examinations	152 (63.3)
Hospital admissions in last 12 months, *n* (%)	(*n* = 238)
Mean (SD)	0.3 (0.6)
Min, Max	0.0, 5.0
Number of hospital admissions in the last 12 months, *n* (%)	(*n* = 238)
0	190 (79.8)
1	37 (15.5)
2	9 (3.8)
≥3	2 (0.8)
Type of inpatient stay: emergency or non-emergency, *n* (%)	(*n* = 48)
Emergency	18 (37.5)
Non-emergency	30 (62.5)
Type of inpatient stay: planned or unplanned, *n* (%)	(*n* = 42)
Planned	19 (45.0)
Unplanned	23 (55.0)
Time spent in hospital, *n* (%)	(*n* = 45)
Day case	30 (66.7)
≥ 1 night	15 (33.3)
Patient in veterinary intensive care, *n* (%)	(*n* = 47)
Yes	8 (17.0)
No	39 (83.0)

CKD, chronic kidney disease; SD, standard deviation.

In a 12-month period, the mean (SD) number of appointments per dog was 3.9 (6.5) and these appointments were most frequently with a veterinarian. Data on diagnostic tests/assessments were available for 253 dogs. The most frequently used tests/assessments to aid CKD diagnosis were serum creatinine (93%), blood urea nitrogen (86%) and complete blood count (75%) (Table 11). Other tests/assessments used to aid diagnosis included serum phosphorous (71%), serum calcium (61%), symmetric dimethylarginine (60%), urine sediment examination (45%), urine dipstick test for protein (40%), urine protein: creatinine ratio (23%), and blood pressure (20%) (Supplementary Table S1). Of 238 responses, the mean (SD) number of tests/assessments used to aid diagnosis of CKD was 8.3 (4.0). Data on tests/assessments used to monitor CKD were available for 240 dogs. A mean (SD) of 7.3 (4.8) tests/assessments were used, with serum creatinine used in 81% of cases, blood urea nitrogen in 73%, and clinical signs and physical examinations in 63% of patients. Other tests/assessments used to monitor included serum phosphorus (61%), symmetric dimethylarginine (50%), serum calcium (49%), blood pressure (25%), and urine protein:creatinine ratio (24%) (Supplementary Table S1).

Mean (SD) hospital admissions per dog in the last 12 months were 0.3 (0.6) as reported for 238 dogs (Table 11). Respondents reported the number of hospital admissions per dog as follows: 0 admissions for 80% of dogs, 1 admission for 16%, 2 admissions for 4%, and ≥ 3 admissions for less than 1%. Most inpatient hospitalizations (*n* = 48) were non-emergencies (63%), although 55% were unplanned (*n* = 42). Veterinarians reported that 67% (*n* = 30) of hospitalizations were day cases that did not require an overnight stay, and 83% (*n* = 39) of patients did not require intensive care during their stay.

## 4 Discussion

To the authors’ knowledge, the Adelphi Real World Canine CKD DSP is the first study to apply this methodology in the veterinary healthcare setting, using RWE to characterize the management of CKD in canines, as well as the impact the disease has on owners and veterinary healthcare resource utilization.

### 4.1 Canine CKD clinical characteristics and disease management

In our study, the average age of dogs at the time of treatment and/or monitoring was 11.4 years, which is characteristic of this disease arising in older patients. Most dogs were diagnosed at IRIS stages I and II, with only 21% reported to have progressed since diagnosis. This is potentially due to CKD being identified at such early stages, allowing for prompt interventions that can slow progression (46, 47). Furthermore, although weight loss was reported in approximately 30% of dogs, most were either at ideal weight or overweight, and had an ideal or over ideal body condition. This could be explained due to most dogs included in the study being at IRIS stage I-II, as significant weight loss typically occurs at more advanced stages of CKD. Common clinical signs reported in this study included polydipsia, polyuria, weight loss, lethargy, and vomiting. This signalment is in line with previous research which showed that affected dogs tend to be diagnosed at an older age (1–3), in early IRIS stages (12), and with clinical manifestations and outcomes similar to those described in the present study (3, 7, 8). The authors recommend further research into the association between weight, body condition and IRIS staging to gain a more comprehensive understanding.

The findings of the current study, which highlight discrepancies in the identification and reporting of clinical signs between veterinarians and dog owners, align with existing research on veterinarian-client interactions. For example, among dogs that were considered dehydrated by veterinarians, only one-quarter were identified with this condition by the owners, highlighting gaps in owner awareness and understanding of clinical signs. This is significant as the failure to identify dehydration can lead to further disease complications such as the worsening of pre-renal azotemia (48), and clinical signs such as polydipsia and lethargy. Additionally, our results also indicate that veterinarians under-reported the number of dogs with clinical signs as well as the number of dogs experiencing problematic clinical signs, with owners reporting vomiting, hyporexia, and diarrhea more frequently than veterinarians. As current guidelines recommend that treatments should be prescribed for clinical signs management (16), it is important for veterinarians to correctly identify patients with these conditions, so that the appropriate treatment can be received. These discrepancies may reflect differences in how clinical signs are observed, prioritized, and discussed by veterinarians and owners. Previous studies have shown that veterinarians often employ a paternalistic communication style in veterinary practice (49), focusing on providing information rather than engaging owners in discussions, which can limit the owners’ ability to recognize subtle clinical signs (50, 51). Effective communication is crucial for improving patient outcomes. Motivational interviewing, a client-centered communication approach, has been recommended to improve alignment between veterinarians and owners by encouraging empathy, active listening and collaboration (52). Incorporating this communication style into veterinary consultation could address gaps in owner education and improve reporting of clinical signs. Taken collectively, these findings highlight the need for further owner education regarding the important clinical signs of CKD, and improved methods of communication between veterinary professionals and owners’ during appointments to improve outcomes for dogs suffering from CKD.

This study also gathered valuable real-world data on the management of canine CKD. Firstly, recommendations provided by veterinary professionals were consistent with those recommended within the literature and current guidelines (14–16). Furthermore, our data showed that owners were implementing the recommendations made by veterinarians, such as the use of a renal diet, which is considered paramount to CKD management (16). A previous study has demonstrated that feeding a renal diet significantly improves survival times and reduces the risk of uremic crises in dogs with CKD (15). However, adherence to renal diets can be challenging due to palatability issues or food refusal, which may limit the effectiveness of this intervention (19). When considering treatments, most dogs in this study had never received treatment for their disease. Of those who were receiving treatment, maropitant – typically used to treat nausea and vomiting – was the most common. Other common treatments were fluid therapy, aluminum hydroxide, and capromorelin. These interventions align with what is recommended in the treatment guidelines (16); however, it is important to note that evidence of efficacy or effectiveness of these interventions is often variable or lacking (20). For example, while controlled clinical studies have established the value of using aluminum hydroxide or other intestinal phosphate-binding agents in dogs with CKD, some aluminum-containing phosphate binders are not always well tolerated and must be given several times a day with meals which may impact on the acceptance of food (20) and exacerbate any adherence issues. Indeed, half of owners in the current study who reported difficulty administering medication, reported that this was due to their dogs’ refusal. To address these challenges, veterinary professionals could consider strategies such as educating owners on medication administration techniques, exploring alternate therapies with less frequent dosing schedules, and using compounded formulations or flavored medications (13, 53).

### 4.2 Experience of owning a dog with CKD

Previous studies have documented that owners often experience heightened stress, symptoms of anxiety and depression, and a reduced quality of life when managing the care of an ill pet (21–23). This burden can be exacerbated by the financial costs of treatment, time demands, and the emotional impacts of the pets’ decline in health (22). In this study, we found that owners experienced the greatest difficulty with their dog not eating (hyporexia). However, this was not identified as a most problematic aspect of care by veterinarians, highlighting a previously unknown aspect of burden for owners of dogs with CKD. Time constraints during veterinary appointments may further intensify this burden by preventing owners from fully discussing their concerns, leaving them feeling unheard and unsupported (49). Limited consultation time can also hinder veterinarians from addressing the emotional aspects of caregiving, such as guilt or anxiety, which are common among pet owners. While veterinarians are trained to focus on clinical outcomes and treatment protocols, these findings emphasize the need to place greater importance on understanding and addressing the emotional challenges faced by pet owners. Improving appointment structures to allow for more comprehensive discussions could help alleviate this burden and provide owners with support during disease management. Overall, this represents an important opportunity for veterinary practices to enhance both clinical care and owner wellbeing.

When work productivity and activity impairment were considered, no significant decline was observed in the surveyed owners. However, it is important to note that the sample sizes for this analysis were low and that further studies with larger sample sizes are recommended to increase our understanding on how CKD in dogs impacts the owners’ ability to work and perform daily activities.

Regarding owner attitudes and perceptions, according to our findings, owning a dog with CKD did not substantially impact on the owners’ dispositions, regardless of IRIS stage. Therefore, it can be considered that the bond an owner has with their dog and the joy associated with it (54, 55) outweighs most of the negative aspects of owning a dog with illness (21–23). Furthermore, previous research has also reported that owners who can anticipate their pet’s death typically view their pet more positively and less negatively (56). As CKD is a progressive and terminal condition in dogs, this may explain why owner’s attitudes toward their pet remain positive in the current study, regardless of IRIS stage. These findings suggest that owners may require additional guidance and support with managing challenging clinical signs, and further research within this topic may provide additional insights into the best approach to support the owners of these patients.

### 4.3 Veterinary healthcare utilization

The utilization of veterinary healthcare resources for CKD primarily occurred in general veterinary practices, with most appointments taking place in this setting. Irrespective of appointment location, veterinary healthcare utilization was evident, with an average of 3.9 appointments and 7.3 tests administered per dog over a 12-month period. According to our findings, the number of hospitalizations for CKD was low, with most dogs not requiring inpatient care. However, it is important to note that the majority of dogs were diagnosed at IRIS stage I-II and were IRIS stage I-II at the time of survey, so therefore may not have reached the stage of disease where hospitalization was necessary. Hospitalization is typically reserved for stabilizing clinical signs, managing complications such as dehydration or uremia, and providing supportive care (4). A previous study demonstrated that all acute cases of CKD required hospitalization, with a median hospitalization time of 5 days, underscoring the need for inpatient care during acute exacerbations or in advanced stages of disease (4). However, to reduce the likelihood of hospitalization in dogs with CKD, proactive management strategies are essential. These include implementing dietary changes, ensuring consistent hydration, scheduling regular veterinary check-ups to allow for the early detection of complications, and educating owners on the warning signs of deterioration (6, 16). Therefore, by prioritizing proactive care and early intervention, veterinary professionals and pet owners can work together to mitigate the risks associated with CKD, reduce the need for hospitalization, and alleviate the burden on the veterinary healthcare sector.

## 5 Strengths and limitations

This study had a number of strengths and limitations. First of all, although the eligibility criteria for participating veterinarians were broad and inclusive, their enrollment was influenced by willingness to complete the survey, as is the case in all studies of this nature. Additional research with a larger sample would be useful to explore relevant associations with WPAI and IRIS stage identified in this study and help to further understanding of the impact of owning a dog with CKD. The cross-sectional design of this study prevents any conclusions about causal relationships, even though identification of significant associations was possible. While recall bias is a common limitation of surveys, the data for these analyses were collected at the time of each patient’s appointment, which is expected to reduce this likelihood. In addition, veterinarians had access to patient medical records for data extraction. Despite such limitations, real-world studies play an important part in highlighting areas of concern that are not addressed in clinical trials, with the data in this study representative of current clinical practice at the time the survey was conducted.

## 6 Conclusion

In conclusion, RWD has the power to overcome a lack of external validity in clinical trials and, ultimately, enhance the management of a disease in a clinical setting. There were notable differences between veterinarians and dog owners in identifying the clinical signs of CKD, highlighting essential areas for improvement in owner awareness and education and veterinarian-owner communication. Caring for a dog with CKD did not substantially influence the owners’ attitudes toward their dog, regardless of disease stage, reflecting the strong emotional connection owners form with their pets. As a first-of-its kind animal RWE study using the approved DSP methodology, this study highlights the value of gathering RWD. The insights gained from this research will enhance our understanding of CKD in dogs, as well as the impact the condition has on owners, ultimately guiding improved management strategies for both veterinarians and pet owners.

## Data Availability

The datasets presented in this article are not readily available because all data, i.e., methodology, materials, data and data analysis, that support the findings of this survey are the intellectual property of Adelphi Real World. Requests to access the datasets should be directed to James Jackson, james.jackson@adelphigroup.com.
