# Supplementary material for: Replicating the real-world evidence methods available in human health to assess burden and outcomes for dogs with chronic kidney disease, their owners, and the veterinary healthcare system in the United States of America
Source: Front Vet Sci. 2025 Feb 21;12:1502933. doi: 10.3389/fvets.2025.1502933 (PMC11886590; doi:10.3389/fvets.2025.1502933)
Supplement: Supplementary file 1 [file Table_1.DOCX]

**Supplementary Table 1.** Veterinarian-reported Additional Data

| **Veterinarian-reported additional data** | **Total Canine CKD Patients** |
| --- | --- |
| **Overall veterinarian-reported clinical signs, n (%)**  Increased/excessive water consumption  Increased/excessive urination  Unexplained weight loss  Halitosis  Weakness/fatigue  Lethargy  Dehydration  Anorexia  Hyporexia  Depression  Unkempt appearance  Vomiting  Gastroenteritis  Problems with vision  No clinical signs  Diarrhea  Pale gums  Urinary tract infection  Other  Blood in urine  Osteoporosis  Mouth ulcers | **(n=253)**  107 (42.3)  94 (37.2)  64 (25.3)  61 (24.1)  60 (23.7)  57 (22.5)  41 (16.2)  39 (15.4)  38 (15.0)  37 (14.6)  32 (12.6)  31 (12.3)  22 (8.7)  17 (6.7)  16 (6.3)  11 (4.3)  11 (4.3)  8 (3.2)  6 (2.4)  4 (1.6)  3 (1.2)  1 (0.4) |
| **Overall veterinarian-recommended changes, n (%)**  Veterinary prescribed renal diet  Increase water intake  Add water to kibble  Avoid high sodium foods  Avoid high phosphorus foods  Reach/maintain a healthy weight  Use omega-3 supplements  Reduce protein consumption  Use food with higher quality protein but in lower quantity  Discontinue nephrotoxic drugs  Feed more frequently  No recommended changes  Use a home prepared renal diet  Use a commercial renal diet  Start/increase exercise activity  Walking the dog less frequently  Use a commercial senior diet  Other lifestyle change  Other nutrition change | **(n=253)**  186 (73.5)  121 (47.8)  85 (33.6)  82 (32.4)  75 (29.6)  75 (29.6)  65 (25.7)  60 (23.7)  40 (15.8)  29 (11.5)  26 (10.3)  19 (7.5)  17 (6.7)  15 (5.9)  12 (4.7)  11 (4.3)  8 (3.2)  5 (2.0)  5 (2.0) |
| **Top 15 tests used to aid diagnosis of CKD, n (%)**  Serum creatinine  Blood urea nitrogen  Complete blood count  Serum phosphorous  Urine specific gravity  Serum potassium  Clinical signs and physical examinations  Serum calcium  Symmetric Dimethylarginine  Urine sediment examination  Packed cell volume  Urine dipstick test for protein  Urine protein: creatinine ratio  Blood pressure (systolic/diastolic/mean arterial pressure)  Abdominal ultrasound | **(n=253)**  234 (92.5)  218 (86.2)  190 (75.1)  180 (71.1)  169 (66.8)  159 (62.8)  157 (62.1)  153 (60.5)  152 (60.1)  113 (44.7)  110 (43.5)  100 (39.5)  57 (22.5)  50 (19.8)  30 (11.9) |
| **Top 15 tests used to monitor CKD, n (%)**  Serum creatinine  Blood urea nitrogen  Clinical signs and physical examinations  Serum phosphorous  Complete blood count  Serum potassium  Urine specific gravity  Symmetric Dimethylarginine  Serum calcium  Packed cell volume  Urine sediment examination  Urine dipstick test for protein  Blood pressure (systolic/diastolic/mean arterial pressure)  Urine protein: creatinine ratio  Serum ionized calcium | **(n=240)**  194 (80.8)  176 (73.3)  152 (63.3)  146 (60.8)  139 (57.9)  129 (53.8)  121 (50.4)  119 (49.6)  118 (49.2)  93 (38.8)  88 (36.7)  82 (34.2)  59 (24.6)  57 (23.8)  21 (8.8) |

CKD, chronic kidney disease; SD, standard deviation
